# Evolving role of 2B4/CD244 in T and NK cell responses during virus infection

**DOI:** 10.3389/fimmu.2012.00377

**Published:** 2012-12-11

**Authors:** Stephen N. Waggoner, Vinay Kumar

**Affiliations:** ^1^Department of Pathology, University of Massachusetts Medical SchoolWorcester, MA, USA; ^2^Program in Immunology and Virology, University of Massachusetts Medical SchoolWorcester, MA, USA; ^3^Department of Pathology, University of ChicagoChicago, IL, USA

**Keywords:** NK cells, T cells, virus infection, XLP, SLAM receptors, 2B4

## Abstract

The signaling lymphocyte activation molecule (SLAM) family receptor, 2B4/CD244, was first implicated in anti-viral immunity by the discovery that mutations of the SLAM-associated protein, SAP/SH2D1A, impaired 2B4-dependent stimulation of T and natural killer (NK) cell anti-viral functions in X-linked lymphoproliferative syndrome patients with uncontrolled Epstein–Barr virus infections. Engagement of 2B4 has been variably shown to either activate or inhibit lymphocytes which express this receptor. While SAP expression is required for stimulatory functions of 2B4 on lymphocytes, it remains unclear whether inhibitory signals derived from 2B4 can predominate even in the presence of SAP. Regardless, mounting evidence suggests that 2B4 expression by NK and CD8 T cells is altered by virus infection in mice as well as in humans, and 2B4-mediated signaling may be an important determinant of effective immune control of chronic virus infections. In this review, recent findings regarding the expression and function of 2B4 as well as SAP on T and NK cells during virus infection is discussed, with a focus on the role of 2B4–CD48 interactions in crosstalk between innate and adaptive immunity.

## INTRODUCTION

Immunity to virus infection involves the complex interplay of many different leukocytes, including natural killer (NK) cells (Bukowski et al., 1983; Biron et al., 1989) and CD8 T cells (Zinkernagel and Welsh, 1976). Pro-inflammatory cytokines, including type I interferon (IFN), can stimulate the activation and proliferation of these cytolytic and cytokine-producing effector cells. Moreover, while CD8 T cell responses are induced by virus-derived peptides in the context of class I major histocompatibility complex (MHC) molecules, NK cell activity can be provoked by interactions of stimulatory NK cell receptors (NKR) such as Ly49H with virally encoded proteins (Brown et al., 2001; Daniels et al., 2001; Lee et al., 2001; Arase et al., 2002; Smith et al., 2002).

Natural killer cell anti-viral function is further regulated by a variety of activating and inhibitory receptors that engage stress-induced proteins (Raulet, 2003) or self-specific MHC antigens (Brutkiewicz and Welsh, 1995) on virus-infected cells. Notably, expression of some NKR is also up-regulated on activated T cells and this expression is often sustained at a high level on virus-specific T cells that have lost functionality or become exhausted in the context of chronic virus infection (Vivier and Anfossi, 2004; Crawford and Wherry, 2009). One particular NKR, 2B4 (CD244/*slamf4*), contributes to the regulation of both NK cell anti-viral activity and virus-specific CD8 T cell functionality (McNerney et al., 2005b), and has recently been implicated in NK cell regulation of virus-specific T cell responses (Waggoner et al., 2010).

## SLAM FAMILY RECEPTORS IN VIRUS INFECTION

2B4 is a member of the signaling lymphocyte activation molecule (SLAM) family of CD2-related receptors, which includes SLAM (*Cd150/slamf1*), CD48 (*slamf2*), Ly9 (CD229/*slamf3*), CD84 (*slamf5*), NK-T-B-antigen (NTB-A in human, Ly108 in mouse, CD352/*slamf6*), and CD2-like receptor activating cytotoxic cells (CRACC/CS1/CD319/*slamf7*; Cannons et al., 2011). Each of these receptors, except CD48, encodes two or more cytoplasmic immunoreceptor tyrosine-based switch motifs (ITSM). Tyrosine phosphorylation of this motif facilitates binding of SLAM-associated protein (SAP) family adaptors SAP (*sh2d1a*), EWS–Fli1-activated transcript-2 (EAT-2/*sh2d1b*), and EAT-2-related transducer (ERT/*sh2d1c*; Veillette et al., 2009). Although most SLAM family receptors engage in homotypic interactions, 2B4 interacts with CD48.

A number of SLAM family receptors are implicated in the pathogenesis of virus infections. The prototypical family member, SLAM, is a cellular receptor for measles virus (Tatsuo et al., 2000). CD48 is highly up-regulated on the surface of Epstein–Barr virus (EBV)-infected lymphoblasts and may contribute to viral trafficking (Thorley-Lawson et al., 1982). The UL7 protein of human cytomegalovirus contains an immunoglobulin-like domain exhibiting remarkable sequence similarity to Ly9/CD229 (Engel et al., 2011). Expression of UL7 in myeloid cells attenuated pro-inflammatory cytokine production.

In contrast to SLAM receptors that are exploited by viruses, NTB-A and 2B4 contribute to effector cell-mediated killing of virus-infected cells. Co-engagement of NTB-A and NKG2D on human NK cells promotes release of lytic granules and lysis of HIV-infected CD4 T cells (Ward et al., 2007). However, HIV-1 has evolved mechanisms to evade this SLAM receptor recognition through Vpu-mediated down-modulation of NTB-A expression on infected CD4 T cells (Shah et al., 2010). Thus, some SLAM receptors are sufficiently important to the host anti-viral response that viruses may encode proteins to negate their functions.

## SAP FAMILY ADAPTORS AND XLP

Interactions between the ITSM motif of SLAM family receptors and the adaptor protein SAP enables recruitment of the Src family tyrosine kinase Fyn, which facilitates downstream signaling to modulate effector functionality (Veillette, 2006). EAT-2 and ERT serve a similar but mechanistically distinct role (Cannons et al., 2011).

The SH2D1A gene that encodes SAP is mutated in patients with X-linked lymphoproliferative (XLP) disease such that the SAP polypeptide is absent or dysfunctional (Coffey et al., 1998). These individuals often present with uncontrolled EBV-induced infectious mononucleosis, characterized by lymphoproliferation, organ failure and death. Infection of *sh2d1a*-deficient mice with lymphocytic choriomeningitis virus (LCMV), *Toxoplasma gondii*, or murine gammaherpesvirus-68 recapitulates many of the disease phenotypes observed in EBV-infected XLP patients, including hyperproliferation of CD8 T cells (Czar et al., 2001; Wu et al., 2001; Yin et al., 2003; Chen et al., 2005) and fatal immunopathology (Crotty et al., 2006). SAP-deficiency also causes defects in long-term humoral immunity (Czar et al., 2001; Crotty et al., 2003; Malbran et al., 2004) and NKT cell development (Chung et al., 2005; Nichols et al., 2005; Pasquier et al., 2005).

Genetic deficiency of any single SLAM family receptor can recapitulate only a subset of the pathologies of SAP-deficiency and XLP disease (Cannons et al., 2011). The defects in humoral immunity are associated with changes in T cell production of cytokines (Ma et al., 2005; Yusuf et al., 2010), including IL-10, and destabilization of interactions between CD4 T cells and cognate B cells (Qi et al., 2008). CD84 and Ly108 play important roles in the conjugation of follicular CD4 T cells and germinal center B cells (Cannons et al., 2010). The defect in the NKT cell lineage in the absence of SAP can be elicited by combined deletion of SLAM and Ly108, suggesting that these receptors are important in the development of NKT cells (Griewank et al., 2007). Furthermore, inhibitory signals derived from Ly108 engagement in the absence of SAP substantially impaired NKT cell development (Kageyama et al., 2012).

A remarkable facet of XLP is the specific sensitivity of these patients to infections with EBV. NK and CD8 T cells from these individuals have an impaired capacity to lyse EBV-infected B cells (Parolini et al., 2000; Bottino et al., 2001; Sharifi et al., 2004; Dupre et al., 2005; Hislop et al., 2010). Among the different SLAM receptors expressed by NK and CD8 T cells, NTB-A and 2B4 substantially contribute to the cytolytic defects of these effectors in XLP (Nakajima et al., 2000; Parolini et al., 2000; Tangye et al., 2000b; Bottino et al., 2001). Analysis of female XLP carriers with heterozygous expression of SAP revealed that while memory CD8 T cells specific for cytomegalovirus or influenza can be SAP-expressing or SAP-deficient, EBV-specific T cells are exclusively SAP+ due to NTB-A- and 2B4-mediated inhibitory signaling in the absence of SAP when ligands of these SLAM receptors are engaged on antigen-bearing B cells (Palendira et al., 2011). Thus, in the context of SAP-deficiency, 2B4 and NTB-A mediate potent inhibition of effector cell cytotoxic function.

## THE DUAL-FUNCTION RECEPTOR, 2B4/CD244

2B4 is expressed by all NK cells, γδ T cells, basophils, and monocytes as well as a subset of memory-phenotype CD8^+^ αβ T cells (McNerney et al., 2005b). It is currently well accepted that engagement of 2B4 by CD48 can mediate activating as well as inhibitory signals, although the nature of functional dichotomy is a contentious issue. In humans, ligation of 2B4 with specific antibodies or CD48-expressing target cells provides an activating signal for NK cells (Tangye et al., 2000a). In XLP patients who lack functional SAP protein, 2B4 ligation can inhibit NK and CD8 T cell function (Nakajima et al., 2000; Parolini et al., 2000; Tangye et al., 2000b).

**FIGURE 1:**
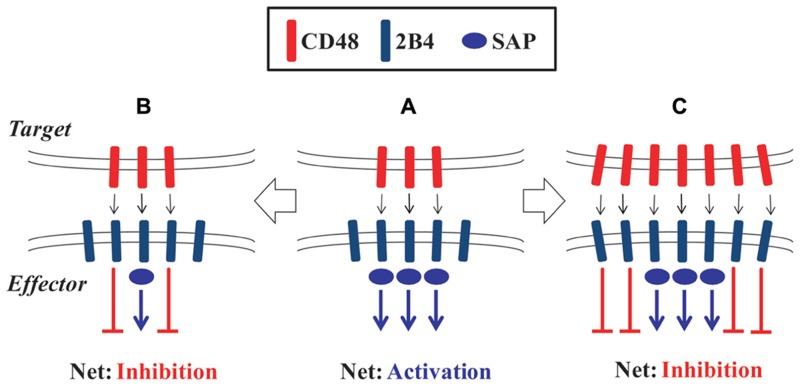
**Molecular model of the dual-function of 2B4**. **(A)** Engagement of 2B4 by CD48 in the presence of sufficient SAP protein leads to activation of NK or CD8 T cell effector cells. **(B)** Insufficient levels of SAP protein leads to predominantly inhibitory signals downstream of 2B4 on effector cells. **(C)** Increased ligand density or receptor expression can also make SAP expression limiting and result in inhibition of effector cell function.

The understanding of the dual nature of 2B4 signaling has been complicated by studies in mice. In contrast to full-length 2B4 which bears four ITSM motifs, alternative splicing in mice generates a short form of 2B4 that contains only one ITSM (Stepp et al., 1999). Whereas overexpression of full-length 2B4 impaired NK cell cytotoxicity, the short form of 2B4 facilitated redirected lysis of tumor cells (Schatzle et al., 1999). Two isoforms of human 2B4 that differ in a small portion of the extracellular domain were also described, where one facilitated lysis of CD48-expressing target cells and the other did not (Mathew et al., 2009). *In vitro* studies have provided conflicting results regarding the effect of 2B4 ligation on murine NK and CD8 T cells, with both activating and inhibitory roles described (Kubin et al., 1999; Kambayashi et al., 2001; Lee et al., 2003; Assarsson et al., 2004; Mooney et al., 2004). Mutual expression of both 2B4 and CD48 by the effector and target cells in those assays could facilitate bi-directional signaling as well as interactions between 2B4 and CD48 expressed on neighboring effector cells (Kambayashi et al., 2001; Lee et al., 2003). Kumar and colleagues (Chlewicki et al., 2008) have proposed that the dual-function of 2B4 is dynamically regulated by the ligand density of CD48, 2B4 receptor expression levels, and availability of intracellular SAP protein in NK cells (**Figure 1**). Importantly, inhibition of NK cell lysis of self within the hematopoietic compartment is mediated by both 2B4 and MHC class I receptors in a non-redundant fashion (McNerney et al., 2005a). Loss of either 2B4- or MHC-mediated inhibition permitted a partial increase in NK cell lysis, whereas loss of ligands for both systems resulted in greatly elevated NK cell lysis of target cells.

## CONTROL OF NK CELL FUNCTION BY 2B4

**FIGURE 2:**
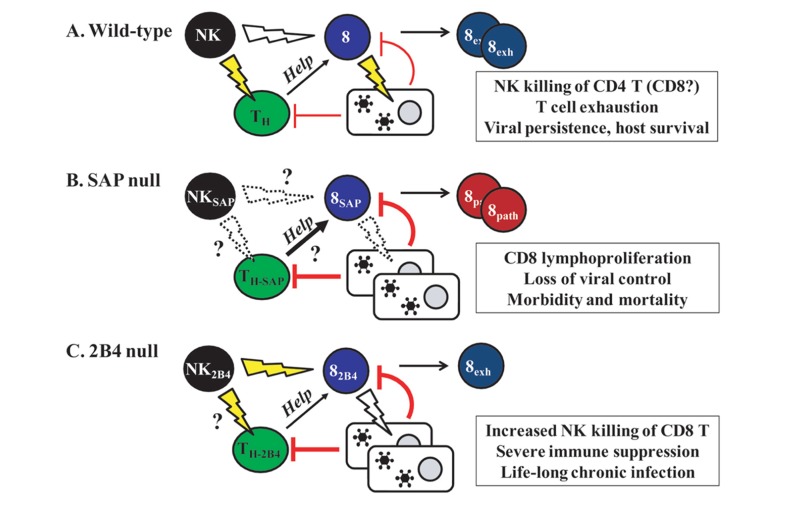
**Modeling the consequences of 2B4 and SAP expression in immunity to persistent virus infection in mice**.**(A)** In a wild-type scenario, virus infection may trigger NK cell lysis of activated CD4 T helper cells and to a lesser extent activated CD8 T cells. This results in less help from CD4 T cells, thereby favoring a loss of CD8 T cell anti-viral function in the context of high levels of replicating virus. Exhaustion of CD8 T cells permits viral persistence with limited immunopathology. **(B)** In the absence of SAP, NK and CD8 T cells exhibit an impaired ability to lyse virus-infected cells, resulting in a loss of viral control. Moreover, we postulate that the immunoregulatory lysis of activated T cells by NK cells may be suppressed in the absence of SAP, thereby contributing to the exaggerated expansion of CD8 T cells and associated fatal immune pathology observed in XLP. **(C)** NK cell lysis of CD8 T cells is enhanced in the absence of 2B4. This causes a severe immune suppression in the context of high viral load, which in turn results in a life-long chronic infection and diminished T cell-mediated tissue damage.

Given that 2B4 could mediate activation of NK and CD8 T cells, it was somewhat surprising that the generation of 2B4-deficient mice underscored an important inhibitory role for murine 2B4 (Lee et al., 2004; Vaidya et al., 2005). 2B4-deficient NK cells demonstrated an enhanced capacity to kill CD48-expressing target cells *in vitro* and *in vivo *(Lee et al., 2004; Vaidya et al., 2005). In the absence of 2B4, NK cells not only lyse hematopoietic tumor cells more efficiently but also begin to target other NK cells (Taniguchi et al., 2007) as well as activated CD8 T cells (Waggoner et al., 2010), suggesting that 2B4 is involved in the maintenance of self-tolerance (McNerney et al., 2005a). While 2B4-deficient mice have strengthened the assertion that 2B4 is an important inhibitor of NK cell cytotoxic function (**Figure 2A**), no 2B4 deficiencies have been identified in humans, and it is unclear whether ablation of 2B4 expression on human NK cells would produce similar effects to those observed in mice.

Expression of 2B4 is dynamically regulated on human NK cells during virus infection. One study found that 2B4 expression on NK cells was transiently reduced after HIV infection (Ostrowski et al., 2005). Likewise, 2B4 expression was reduced on NK cells in hepatitis C virus (HCV)-infected individuals who demonstrated enhanced control of viral titers after type I IFN therapy (Ahlenstiel et al., 2011). Thus, reduced 2B4 expression on NK cells may be indicative of the direct anti-viral activity of NK cells. Nonetheless, human NK cells up-regulated 2B4 expression and displayed enhanced functional activity following *in vitro* exposure to or intramuscular vaccination with influenza A virus (Jost et al., 2011). In addition, heightened transforming growth factor-beta (TGF-β) expression during the immune tolerant phase of persistent hepatitis B virus (HBV) infection was associated with reduced expression of both 2B4 and SAP by NK cells (Sun et al., 2012). These low levels of 2B4 and SAP were further correlated with impaired cytotoxic and IFN-γ-producing activities of NK cells. Of note, TGF-β has recently been shown to contribute to the immaturity of NK cells and susceptibility to virus infection during murine infancy (Marcoe et al., 2012). Together, these studies support the idea that 2B4 functions as an activating receptor on human NK cells during virus infection.

## EXPRESSION OF 2B4 BY VIRUS-SPECIFIC T CELLS

A subpopulation of CD8 T cells with an activated/memory-phenotype express 2B4, which is postulated to play a co-stimulatory role in T cell activation (Assarsson et al., 2004; Altvater et al., 2009). In the absence of functional SAP (**Figure 2B**), 2B4 can also profoundly impair CD8 T cell cytotoxicity against EBV-infected B cells (Hislop et al., 2010; Palendira et al., 2011). In a similar fashion, human T-lymphotropic virus I (HTLV-I)-specific CD8 T cells express higher levels of both 2B4 and SAP in patients with HTLV-I-associated neurological disease than in asymptomatic carriers of HTLV-I (Enose-Akahata et al., 2009). Antibody-mediated blockade of 2B4 or knockdown of SAP expression impaired degranulation and IFN-γ production by the HTLV-I-specific CD8 T cells which mediate disease. While this seems to indicate a role for 2B4 in stimulating virus-specific CD8 T cell function, sustained overexpression of 2B4 may also contribute to dysfunction of exhausted virus-specific CD8 T cells during chronic infection.

Chronic viral antigen presentation can drive functional exhaustion and even deletion of virus-specific T cells in both humans and mice (Shin and Wherry, 2007). High level expression of 2B4 and other inhibitory receptors has also been observed on exhausted CD8 T cells specific for LCMV (Wherry et al., 2007), HIV (Aldy et al., 2011), HCV (Bengsch et al., 2010), and HBV (Raziorrouh et al., 2010). Blockade of 2B4–CD48 interactions restored murine LCMV- (Blackburn et al., 2009) and human HBV-specific (Raziorrouh et al., 2010) CD8 T cell effector functions during *in vitro* culture. Moreover, 2B4-deficient LCMV-specific CD8 memory transgenic T cells were maintained at greater levels than wild-type controls during clone 13 infection of mice (West et al., 2011). Although these results point to a prominent contribution of 2B4 to functional impairment or deletion of virus-specific CD8 T cells (**Figure 2A**), another study found that cross-linking of 2B4 on HCV-specific CD8 T cells could either inhibit or enhance effector functionality depending on the expression levels of both 2B4 and SAP within T cells (Schlaphoff et al., 2011). In addition, antibody-mediated engagement of 2B4 counteracted PD-1 blockade-induced enhancement of HCV-specific CD8 T cell proliferation during *in vitro* culture of human PBMCs. The complex nature of 2B4/PD-1 mediated control of human T cell exhaustion could potentially be explained by our recent findings regarding the role of 2B4 on NK cells in preventing NK-mediated lysis of activated CD8 T cells (Waggoner et al., 2010).

## ROLE OF 2B4 IN NK–T CELL CROSSTALK AND VIRAL PATHOGENESIS

In mice, NK cells play an important role in restricting virus replication during murine cytomegalovirus (MCMV) infection (Bukowski et al., 1983). NK cells may also contribute to immunity by regulating the magnitude and function of MCMV-specific T cell responses (Bukowski et al., 1984; Su et al., 2001; Robbins et al., 2007; Lee et al., 2009; Andrews et al., 2010; Stadnisky et al., 2011; Mitrovic et al., 2012; Narni-Mancinelli et al., 2012). NK cells could impair T cell responses by restricting antigen presentation (Andrews et al., 2010; Mitrovic et al., 2012) or through production of anti-inflammatory cytokines like IL-10 (Lee et al., 2009). Conversely, other studies reported that NK cells sustained conventional dendritic cell populations to enhance anti-viral T cell responses (Robbins et al., 2007; Stadnisky et al., 2011). However, these immunoregulatory contributions of NK cells are difficult to distinguish from the important role of NK cells in direct control of MCMV replication.

In contrast to MCMV, NK cells are largely dispensable in early control of LCMV infection (Bukowski et al., 1983; Welsh et al., 1991). We and others recently described an important role for NK cells in determination of viral clearance and disease associated with LCMV infection that involves direct lysis of virus-specific T cells (Waggoner et al., 2010, 2012; Lang et al., 2012). During infection with the clone 13 strain of LCMV, NK cells aided in the development of persistent infection rather than fatal immunopathology through perforin-mediated lysis of activated CD4 T cells, a population which acts to sustain function of the virus-specific CD8 T cells that mediate virus clearance and tissue damage (Waggoner et al., 2012). NK cells also regulate CD4 responses during LCMV Armstrong infection of CD8 T cell-deficient mice (Su et al., 2001).

Natural killer cells can also mediate direct lysis of activated CD8 T cells (Rabinovich et al., 2003; Soderquest et al., 2011), but this lysis is much more effective when NK cells are not restrained by 2B4 expression (Waggoner et al., 2010). Through the use of different strains of LCMV and NK cell-deficient mice, Ohashi and colleagues (Lang et al., 2012) also demonstrated that NK cells control viral persistence and immunopathology during LCMV infection, but attributed this regulatory effect to perforin-dependent restriction of CD8 T cell expansion without addressing a role for CD4 T cells. Using a modified *in vivo* cytotoxicity assay, we showed that virus infections and pro-inflammatory stimuli (e.g., polyI:C) triggered a rapid, perforin-dependent lysis of activated, but not naive CD4 T cells (Waggoner et al., 2012). NK cell-dependent elimination of CD8 T cells was limited in these assays, suggesting that activated CD4 and CD8 T differ in their susceptibility to NK cell lysis. Notably, activated CD8 T cells expressed more CD48 than their activated CD4 T cell counterparts during LCMV clone 13 infection (Waggoner et al., 2012), and in the absence of 2B4, NK cells displayed an enhanced ability to lyse activated CD8 T cells (Waggoner et al., 2010). As a consequence of dysregulated NK cell lysis of activated CD8 T cells, 2B4-deficient mice suffered a life-long chronic infection with LCMV clone 13 (**Figure 2C**). Thus, 2B4–CD48 interactions can prevent NK cell lysis of CD8 T cells and the resulting loss of viral control. In combination with the effects of 2B4 expression by either NK cells or CD8 T cells on the individual anti-viral activities of these effector cells described in the preceding sections, the role of 2B4 in NK cell/T cell crosstalk may be important to consider when evaluating the use of antibody-mediated blockade of 2B4–CD48 interactions in the rescue of the exhausted virus-specific T cell response (Blackburn et al., 2009; Schlaphoff et al., 2011).

Of note, spontaneous clearance of HCV infection (Khakoo et al., 2004), delayed progression to AIDS during HIV infection (Martin et al., 2007), and resistance to HIV infection in chronically exposed sex workers (Jennes et al., 2006) have all been associated with expression of inhibitory killer immunoglobulin-like receptor (KIR) and their cognate HLA ligands. One interpretation of this observation is that stronger inhibition of NK cell immunoregulatory activity by an inhibitory KIR, or perhaps an inhibitory SLAM receptor such as 2B4, could enhance the virus-specific T cell response against the virus. In fact, the potency of NK cell responsiveness was inversely correlated with the magnitude of HIV-specific T cell responses in a cohort of elite controllers (Tomescu et al., 2012).

## CONCLUDING REMARKS

Immunity to virus infection often involves NK cells and virus-specific T cells, whose responses must be regulated in order to prevent excessive lymphoproliferation that could lead to immunopathology, autoimmunity, and cancer. Growing evidence suggests NK cells may be one mechanism which restrains potentially pathogenic effector T cell responses and that such immunoregulation may itself be regulated by the SLAM receptor 2B4. Heightened expression of 2B4 on NK and CD8 T cells during persistent virus infection may contribute to viral pathogenesis by regulating the anti-viral cytokine-producing and cytolytic functions of these anti-viral effectors. Interactions between 2B4 and CD48 may also contribute to anti-viral immunity in an apparent indirect fashion by controlling the ability of NK cells to regulate virus-specific T cell responses. Evidence from a number of different virus infections demonstrates that 2B4-mediated signals can contribute in both beneficial and detrimental manners to viral clearance and immune-mediated disease. An improved understanding of how 2B4 regulates both effector cell function and NK cell/T cell crosstalk would facilitate development of a number of translational avenues. For example, blockade of 2B4/CD48 interactions may enhance effector functions of NK or CD8 T cells during chronic infections. However, one must temper this prospect with concerns over whether 2B4 blockade would ultimately impair adaptive immunity by unleashing NK cell responses against T cells. The latter possibility itself has translational potential. An improved understanding of how and to what extent 2B4 regulates NK cell lysis of activated T cells could be used to generate NK cell-based therapeutic strategies for autoimmune diseases where T cells are active mediators of disease.

## Conflict of Interest Statement

The authors declare that the research was conducted in the absence of any commercial or financial relationships that could be construed as a potential conflict of interest.

## Acknowledgments

This work was supported by NIH training grant AI07349 and the Ellison Medical Foundation to Stephen N. Waggoner.

## Abbreviations

EBV: Epstein–Barr virus
HBV: hepatitis B virus
HCV: hepatitis C virus
HTLV-I: human T-lymphotropic virus I
IFN: interferon
ITSM: immunoreceptor tyrosine-based switch motif
KIR: killer immunoglobulin-like receptor
LCMV: lymphocytic choriomeningitis virus
MCMV: murine cytomegalovirus
MHC: major histocompatibility complex
NK: natural killer
NKR: NK cell receptor
NTB-A: NK-T-B-antigen
PD-1: programed death-1
SAP: SLAM-associated protein
SLAM: signaling lymphocyte activation molecule
TGF-β: transforming growth factor-beta
XLP: X-linked lymphoproliferative
